# The Intricate Evolutionary Balance between Transposable Elements and Their Host: Who Will Kick at Goal and Convert the Next Try?

**DOI:** 10.3390/biology11050710

**Published:** 2022-05-06

**Authors:** Marianne Yoth, Silke Jensen, Emilie Brasset

**Affiliations:** iGReD, CNRS, INSERM, Faculté de Médecine, Université Clermont Auvergne, 63000 Clermont-Ferrand, France; marianne.yoth@uca.fr (M.Y.); silke.jensen@uca.fr (S.J.)

**Keywords:** transposable elements, endogenous retroviruses, horizontal transfer, piRNA, piRNA cluster, genomic instability, inheritance

## Abstract

**Simple Summary:**

Transposable elements (TEs) are mobile DNA sequences that can jump from one genomic locus to another and that have colonized the genomes of all living organisms. While TE mobilization is an important source of genomic innovations that greatly contribute to the host species evolution, it is also a major threat to genome integrity that can lead to pathologies. In this review, we discuss how TEs successfully bypass the host silencing machineries to propagate in the host’s genome and how hosts engage in a fightback against TE invasion and propagation. We highlight how TEs and their hosts have been evolving together to achieve a fine balance between transposition activity and repression.

**Abstract:**

Transposable elements (TEs) are mobile DNA sequences that can jump from one genomic locus to another and that have colonized the genomes of all living organisms. TE mobilization and accumulation are an important source of genomic innovations that greatly contribute to the host species evolution. To ensure their maintenance and amplification, TE transposition must occur in the germ cell genome. As TE transposition is also a major threat to genome integrity, the outcome of TE mobility in germ cell genomes could be highly dangerous because such mutations are inheritable. Thus, organisms have developed specialized strategies to protect the genome integrity from TE transposition, particularly in germ cells. Such effective TE silencing, together with ongoing mutations and negative selection, should result in the complete elimination of functional TEs from genomes. However, TEs have developed efficient strategies for their maintenance and spreading in populations, particularly by using horizontal transfer to invade the genome of novel species. Here, we discuss how TEs manage to bypass the host’s silencing machineries to propagate in its genome and how hosts engage in a fightback against TE invasion and propagation. This shows how TEs and their hosts have been evolving together to achieve a fine balance between transposition and repression.

## 1. Introduction

Transposable elements (TEs) are DNA sequences that can move and multiply within the genome by transposition. They were discovered by Barbara McClintock in the maize genome in 1950 [1]. Since then, TEs have been found in all living organisms in which they have been searched for. The TE community is still debating whether there are organisms without TE sequences in their genome. Their amplification within genomes leads to the formation of families of repeated sequences that can be present, for some of them, in thousands of copies spread in all chromosomes. They are currently classified in two categories [2]: (i) class I elements, also referred to as retrotransposons, that transpose through a copy-and-paste mechanism, and (ii) class II elements, referred to as DNA transposons, that transpose through a cut-and-paste mechanism (excision and re-insertion at a new locus). TE transposition is a major source of genetic instability, especially through chromosome breakages and insertions that result in mutations, ectopic recombination, and genomic rearrangements. To preserve genome integrity, TE mobilization is strictly controlled by several elaborate defense mechanisms, such as silencing strategies based on KRAB zinc-finger proteins, small RNAs, DNA methylation, and chromatin modifications [3,4,5,6,7]. Moreover, most transposon sequences accumulate mutations that do not allow them to produce the proteins required for their transposition and that may ultimately lead to elimination of all active copies of that mobile element in a population. The combined actions of silencing, mutations, and elimination should result in the complete elimination of TEs from the genome. Yet, TEs represent a large part of the genome in all organisms (prokaryotes, unicellular and multicellular eukaryotes), ranging from 3% in yeast to 85% in maize [8]. Indeed, it is assumed that TE movement and accumulation are an important source of genomic and epigenomic variations that strongly influence the species evolution and adaptation to changing environments [9,10,11,12]. However, it remains unclear how TEs persist in the genome, reach such high proportions, and expand in all living species, while transposition is strictly controlled. Here, we review the strategies developed by TEs for effective spreading and to bypass the host silencing machinery. We also describe how organisms fight back to protect against TE invasion and to control their propagation.

## 2. Genome Invasion by Transposable Elements: Strategies for Effective Spreading

### 2.1. Horizontal Transfer: TE Propagation between Species

A major step in understanding how TEs might persist over time was the discovery that some TEs can colonize “naive” genomes through horizontal transfer (HT). HT is the transmission of genetic material between closely or distantly related organisms in the absence of reproduction. These events permit the acquisition of exogenous genetic material and, therefore, are responsible for the appearance of genetic novelties. The first evidence of HT involving TEs (Horizontal Transposon Transfer, HTT) in eukaryotes was the HT and subsequent invasion by the *P-element*, a DNA transposon, between two fruit fly species (i.e., from *Drosophila willistoni* to *Drosophila melanogaster*). *P-elements* rapidly spread through natural populations of *D. melanogaster* between 1950 and 1980, and all flies collected in the wild after 1980 have *P-elements,* unlike laboratory strains derived from flies collected before 1950 [13,14,15,16]. The *P-element* in the *D. melanogaster* genome differs by only one nucleotide from that in the *D. willistoni* genome. This demonstrated that the *P-element* found in *D. willistoni* was transferred to *D. melanogaster* some time before 1950. Currently, the number of fully sequenced genomes is sufficiently high to reveal that HTT is a widespread phenomenon in metazoans. For instance, more than 500 putative HTT events have been described between *Drosophila* species [17]. In insects, HTT is not an anecdotic event because up to 24% of all nucleotides of insect genomes might come from HTTs [17]. The same authors showed that DNA transposons transfer horizontally more frequently than retrotransposons. These findings indicate that HTT is a fundamental mechanism implicated in eukaryotic genome evolution. It allows TEs to bypass the host silencing machinery by introduction into naive species that have not yet adapted to silence new TEs.

**Figure 1:**
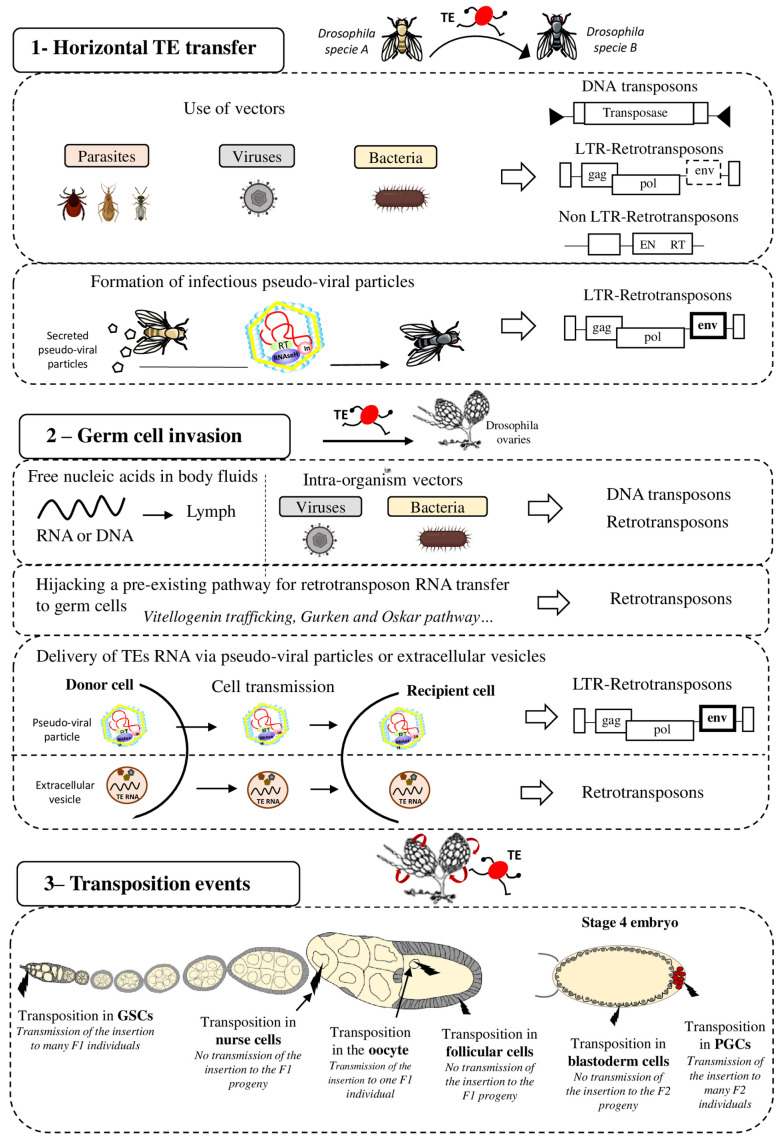
Strategies for effective spreading of transposable elements in the *Drosophila* genome. Transposable elements (TE), DNA transposons and LTR and non-LTR retrotransposons, can colonize “naive” genomes through horizontal transfer (**panel 1**). This may occur via vectors (e.g., parasites, viruses, and bacteria) that transfer genetic content from one organism to another. It has also been proposed that LTR retrotransposons (ERV) can form pseudo-viral particles with infectious properties. Then, invading TEs need to reach the host’s germline (**panel 2**) for vertical transmission to the progeny. To this end, TE sequences (excised DNA or RNA for retrotransposons) might circulate in the blood or be transported to germ cells by intra-organism vectors (viruses, bacteria). Retrotransposons can hijack pre-existing cell pathways, such as the vitellogenin trafficking (*ZAM*), Gurken (*I-element*) or Oskar (*TAHRE*) pathways, to transfer their RNA to germ cells. LTR-retrotransposons (ERV), through the formation of infectious pseudo-viral particles, might also be transferred between cells and/or circulate through extracellular fluids to reach the host’s germline. Extracellular vesicles have also been proposed to be efficient vectors for transferring TEs between host cells The last step for efficient invasion requires transposition in germ cells (**panel 3**) that allows or not (in function of the cell type) the TE transmission and propagation in the specie. GSC = Germinal Stem Cells, PGC = Primordial Germ Cells.

TE mobility and replication characteristics may facilitate the invasion and integration into the host genome. However, the precise mechanisms by which TEs can be shuttled between organisms and the nature of the potential vectors remain speculative (Figure 1 panel 1). It has been suggested that host–parasite interactions favor HTT. For instance, the *P-elements* could have been transmitted from *D. willistoni* to *D. melanogaster* thanks to the mite *Proctolaelaps regalis* [18]. During feeding by piercing and sucking the fly eggs and larvae, this mite might transmit genetic material (e.g., DNA transposons) from one fly to another. Insects, such as wasps or the hemipteran *Rhodnius prolixus*, that feed on the blood of mammals, birds, and reptiles might be involved in HT as vectors (reviewed in [19]). Bacteria and viruses also might be interesting vectors of HTT between species. Indeed, their capacity to transfer DNA and recombine with the host genome might allow them to transport various TE sequences from host to host. Gilbert et al. analyzed 21 genomes of a baculovirus population and demonstrated that a substantial number of TEs from the infected host can transpose into the baculovirus genome [20]. The discovery of a retroposon sequence (Short Interspersed Nucleotide Element, SINE) and its flanking regions coming from the genome of a West African snake (*Echis ocellatus*) in the genome of the taterapox virus (TATV) is another piece of evidence that viruses are frequently used as vectors for HTT [21]. *Wolbachia* is an intracellular parasitic bacterium that infects mainly arthropod species and also some nematodes. This bacterium transfers vertically and horizontally between species and can also transit from cell to cell and infect the host germ cells. Interestingly, many gene transfer events have been detected between *Drosophila* and *Wolbachia*, suggesting that this bacterium is a good candidate vector for HTT between arthropods [22,23]. As a final example, it has been proposed that nematodes may be both great vectors for HTT but also serve as TE reservoirs [24]. Nematodes are ubiquitous organisms and the geographical proximity with many different species increased their chance to participate either as a donor or as a recipient in HTT events. In line with that, many horizontal transfer events involving TEs have been reported to occur between nematodes and unrelated organisms. Future phylogenetic studies will probably reveal many other HTT events involving many different mechanisms and vectors. Combined with geographical and ecological data, these findings will help to unravel the complex and dynamic network of gene transfer and HTT that break down taxon boundaries, and to determine the contribution of ancient HTT events to the evolution of different organisms.

### 2.2. Spread within an Organism and Vertical Transfer to Descendants

#### 2.2.1. Germ Cell Invasion: A Life or Death Issue

After invasion of an organism, TEs must then reach the host’s germ cells, the only cells whose genetic material will be transmitted to the offspring (vertical transmission) (Figure 1 panel 2). Indeed, if transposition occurs only in somatic cells, the horizontally transferred TEs will die with the host and will never spread within that species. Therefore, successful HTT requires the TE integration into the germ cell genome. Although germline and soma are well distinct cell types in animals, HTT is not rare in complex multicellular eukaryotes, showing that genetic material is transferred from soma to germline. It is still unknown when and in which cells during germ cell development TEs transpose and integrate the germline genome after HTT. Indeed, TE sequences must circulate in the body to reach the germline. They may be transported by their own pseudo-viral particles or by vectors, such as *Wolbachia* bacteria, or even in the form of free RNA or DNA [25]. DNA and RNA molecules can circulate in the extracellular body fluids, such as blood, plasma, lymph, saliva, or milk [26]. It has also been proposed that extracellular vesicles (EVs) could be efficient vectors for transferring TEs between host cells [27]. EVs, such as exosomes or microvesicles, are cell-derived vesicles that deliver biological molecules between different cells and cell types in organisms [28]. LINE1 retrotransposon RNAs have been detected in EVs isolated from cells expressing LINE1 active elements, and these EVs can then deliver LINE1 RNA to recipient cells. Thus, EVs could deliver retrotransposon RNA to neighboring and distant cells, potentially permitting germ cell invasion.

Once the TE reaches the germ cells, transposition into the germ cell genome is the next critical step to ensure its inheritance. It is important to note that gonads are made of different cell types and that transposition can occur at different stages of gametogenesis and in the different cell types (Figure 1 panel 3). The consequences of transposition depend on the type of gonad cells in which transposition occurs. For instance, in *Drosophila* embryos, Primordial Germ Cells (PGCs) give rise to all Germinal Stem Cells (GSCs) present in adult gonads. In adults, GSCs divide asymmetrically to produce one daughter GSC and one cystoblast. The cystoblast begins to differentiate and undergoes four rounds of mitotic divisions to form a cyst of 16 germinal cells. Most of them become nurse cells and only one will differentiate into the future oocyte. The oocyte is the only germ cell that will progress through meiosis and will be fertilized. Nurse cells do not transfer their genetic content to the progeny. Consequently, transposition in nurse cells is not expected to be beneficial to the TE. However, these cells show a high level of polyploidy and produce huge RNA quantities. For TEs, nurse cell invasion could be an intermediate and suitable strategy to reach the oocyte, especially if they transpose through an RNA intermediate, as retrotransposons do. Once inserted in the DNA of nurse cells, TEs might be expressed and produce a huge quantity of RNA that will then be transmitted to the oocyte when nurse cells dump their content into the oocyte. In agreement, the *I-element* (a *Drosophila* retrotransposon) is expressed only in nurse cells and then *I-element* RNA transits to the oocyte to integrate its genome [29]. The RNAs of other TEs (*HMS-Beagle, 3S18, Blood, Max, TAHRE*, *Burdock*, and *HeT-A*) also can target the oocyte [29,30,31]. TEs have developed a variety of strategies to optimize their transfer to specific target cells, such as the oocyte, particularly by hijacking pre-existing pathways in the host organism. For instance, *I-elements* target the oocyte nucleus by exploiting the host transport machinery of *gurken* mRNA [32]. *I-element* transcripts contain a small loop of secondary structure that resembles a structure present in *gurken* mRNA. This loop represents a consensus signal for targeting RNAs to the oocyte nucleus by dynein-mediated transport. Similarly, *TAHRE* transcripts migrate to the oocyte germ plasm by mimicking *oskar* RNAs and engaging the Staufen-dependent active transport machinery [30]. Once in the oocyte, TE RNA can be reverse transcribed before integration in the oocyte DNA. However, the highly condensed oocyte genome makes transposition events difficult and unlikely in this cell type. Yet, RNAs present in the ooplasm are transmitted to the embryo. Thus, transposition can also occur in the embryo, particularly in its PGCs. TE integration in the embryo PGCs is particularly advantageous because the new insertions will be inherited by the next generation through the myriad of gametes produced. For example, *P-elements* can transpose to PGCs in embryos and in GSCs in adult ovaries, and this could explain their rapid propagation in *D. melanogaster* populations [33].

#### 2.2.2. Last Step for an Efficient Invasion: Transposition and Fixation in Target Cells

Transposition in the germ cell genome is crucial for TE propagation in a population because it allows the vertical transmission of new insertions. However, the transposition rate is very low. For example, 10^−4^ transposition events per TE copy per generation occur in *Drosophila* natural and laboratory populations [34,35,36]. Although the transposition rate can be higher in conditions of environmental or genomic stress, this is not sufficient to explain how genome invasion is quickly observed after HTT. According to the model proposed by Le Rouzic and Capy, following invasion by HT, an initial transposition burst occurs [37] that leads to TE accumulation in the genome before the induction of an adaptive response by the host to control transposition. Unfortunately, it is almost impossible to observe transposition bursts in real time because they can be very fast. However, the rapid invasion of *P-elements* in natural *D. melanogaster* populations following HTT strongly suggests that the rate of *P-element* transposition must have been very high at one point. Interestingly, Kofler and al. have demonstrated that *D. simulans* populations that are at their starting point of *P-element* invasion have a high *P-element* transposition rate during the first time of invasion [38]. A similar observation has been made analyzing the transposition dynamics of the *mariner* DNA transposon after its introduction into *D. melanogaster* populations containing no active *mariner* [39]. The high transposition rate of the introduced *mariner* element leads to the invasion of the population and the colonization of the genome. Finally, by artificially introducing TEs in naive species to mimic HTT, it has been demonstrated that the introduction of DNA transposons (e.g., *Tc1*, *hAT*, and *PiggyBag*) in the genome of species that belong to different kingdoms or domains of life leads to high transposition rates that depend on the TE class and expression pattern [40]. It is important to note that the capacity to transpose may vary among TEs. As an example, it has been proposed that the great success of DNA transposons to transfer horizontally compared to retrotransposons could be explained by their “blurry promoters” [41]. Actually, DNA transposon expression shows very low dependence on host factors; these TEs are more broadly expressed in diverse organisms allowing them to transpose in a large panel of hosts. Another explanation could be that DNA intermediate molecules of DNA transposons are more stable than RNA intermediate molecules used for retrotransposon transposition.

At the end, TEs that can invade genomes are certainly those that can implement an efficient invasion strategy and transposition mechanisms. Moreover, insertions that are neutral or that increase the host fitness have higher chances to be fixed in a population [42]. However, a quantitative population genetics model showed that a TE may persist as long as its deleterious effect on the host is lower than the advantage of transposition explaining that even TEs with negative fitness effects may spread in populations [43,44].

This led to the conclusion that selective pressure is exerted on TEs during the first steps of invasion, depending on their burst capacity and the effect of TE insertions on population fitness. However, there is a “common advantage” for both host and TEs in limiting massive transposition in the whole organism. In fact, transposition in somatic cells can be deleterious by creating detrimental mutations that lead to the host death and concomitantly to the TE disappearance. From this point of view, TEs resemble viruses: they must multiply and spread, but the host also must survive. It has been hypothesized that several TEs, such as the *P-element* and the *I-element*, have developed the ability to be expressed only in germ cells [45,46,47]. This avoids the deleterious effects of mutations in somatic cells and ensures the transmission of new TE insertions to the progeny. However, transposition in germ cells could also have dangerous outcomes because these mutations are inheritable. Transposition in PGCs (the precursors of all germ cells) is certainly very efficient for TE invasion, but it is highly risky. Indeed, germline transpositions may induce infertility and also deleterious inheritable pathology-causing mutations that potentially endanger the species’ survival. One of the best described deleterious effects of TE transposition in the germline concerns the massive mobilization of TEs that might have contributed to the extinction of Wrangel island mammoths. This small population of mammoths accumulated a large number of detrimental mutations, including deletions and point mutations, and also many TE sequences. This suggests high TE activity in the mammoth germline that led to a very high number of heritable mutations. This high transposition rate in the germline may have contributed, with other factors, to the extinction of this small endangered population [48].

Therefore, TE transposition must be controlled in all cell types to limit its negative effects on the host and its progeny. On the other hand, a too strict control can cause the TE loss from the host genome and deprive the organism of an important source of genetic diversity. For example, *Spermophilus tridecemlineatus* is a rodent in which transposon activity has declined over at least 4 million years. Its genome does not harbor any recent TE, Long Interspersed Nucleotide Element (LINE), SINE, retrotransposon with long terminal repeats (LTR), or DNA transposon activity. Moreover, no functional TE copy is found in the genome of this species because all harbor a huge number of mutations. This is explained by the strong TE silencing, leading to complete inhibition of TE mobilization [49]. To be conserved in a genome, a minimum of transposition is required. Interestingly, in several eukaryotes, temporary relaxation of the TE silencing machinery has been observed in the germline and its associated cells. For instance, during *Drosophila* early oogenesis, there is a short spatiotemporal window when the piRNA pathway seems to be less efficient and at least some TEs might escape the host control. It has been proposed that this window, termed the ‘Piwiless pocket’, allows the insertion of new TEs in the developing germline genome [50,51,52]. In mammals, transient TE relaxation during germ cell development has been observed mainly during epigenetic reprogramming periods [5,53]. Specifically, during the first wave of global reprograming that occurs following fertilization, 10% of the transcriptome in 2-cell stage mouse embryos is made of specific TE transcripts, including transcripts from the *MuERV* retroelement [54,55,56]. The second reprogramming wave occurs in PGCs of the developing mouse embryo. Although no general transcriptional burst has been observed for TEs at this step, some specific TE transcripts (i.e., *LINE1* transcripts) are overrepresented [53], reviewed in [57]. The presence of such spatiotemporal windows during germline development in which TE control is weaker could help to explain the very successful genome invasion by TEs. Once settled in the germline genome, new TE insertions are then vertically transmitted, like any other DNA sequence.

### 2.3. Retrotransposons: A Formidable Capacity of Propagation

#### 2.3.1. Retrotransposons Can Do Intercellular Transposition

Retrotransposons have evolved in a variety of organisms, from protozoa to humans, and display outstanding capacities of rapid invasion and propagation. There are two types of retrotransposons: with and without LTR. Non-LTR retrotransposons lack LTR and have generally two open reading frames of which one encodes a reverse transcriptase and an endonuclease. SINEs do not encode a functional reverse transcriptase and are non-autonomous elements because their transposition relies on enzymes encoded by other non-LTR retrotransposons: the LINEs. Thus, SINEs cannot colonize a naive genome after HT if the genome does not have a corresponding element for trans-complementation.

This part of the review will focus on the other retrotransposon group: LTR retrotransposons. LTR retrotransposons resemble retroviral proviruses. Indeed, they have LTRs at each extremity and open reading frames equivalent to the *gag* and *pol* genes. *Gag* encodes a structural protein involved in the formation of virus-like particles. *Pol* encodes proteins that are necessary for transposition mechanisms: an integrase, a RNase H, a protease, and a reverse transcriptase. Some LTR retrotransposons also harbor the envelope gene (*env*) that encodes a viral surface glycoprotein, and they are called endogenous retroviruses (ERV). ERVs are assumed to be derived from past retroviral infections that have been integrated as permanent residents in host genomes. Like retroviruses, most ERVs can form virus-like particles (VLP). The Env protein interacts with target host cell receptors and allows the fusion of the VLP with the target cell membrane and ERV propagation between cells. ERVs make up approximatively 10% of the mouse, rat, and human genomes and they have been extensively studied in *Drosophila* [8]. In the *Drosophila* genome, many ERVs copies are present, such as the very diverse *Gypsy-like* elements including *ZAM* for instance. These TEs can form VLPs and infect neighboring cells [58,59].

#### 2.3.2. Horizontal Transfer of Retrotransposons: Do They Really Need Vectors?

It has been hypothesized that VLPs produced by ERVs can propagate between organisms, like retrovirus particles, without vectors (Figure 1 panel 1). In this case, these particles could be infectious. VLPs of the *Gyspy* ERV have been found as extracellular particles in the medium in which *D. melanogaster* follicular cells were cultured. This means that *Gypsy* VLPs are secreted by cells [60]. Moreover, these VLPs can infect cultured cells belonging to another *Drosophila* species: *Drosophila hydei.* Interestingly, a recent study showed that *Gypsy* also transits between cells that are not in contact [61]. Furthermore, experiments in which flies were grown on medium containing crushed pupae that produced *Gypsy* VLPs suggested a possible HTT via food: these flies became infected by *Gypsy* [62]. Once transmitted to a new individual by HTT, retrotransposons could use their retroviral properties to propagate between cells and through body fluids to reach the germline, ensuring their spread in that species.

#### 2.3.3. Drosophila Germ Cell Invasion by Retrotransposons

Most TEs can insert in the germline by being active directly in these cells. On the other hand, ERVs do not seem to be expressed in germ cells. In *Drosophila* ovaries, when the pathway regulating TEs is abolished in all cell types, ERVs are only expressed in a patch of somatic cells that are called follicular cells and that surround germ cells [63,64,65,66,67,68]. Indeed, as described for gene transcription, TE transcription requires transcription factors that are present in specific cell subsets [69,70]. For instance, *ZAM* retrotransposon expression requires the presence of Pointed 2, a transcription factor only expressed in a patch of follicular cells [70]. This means that ERV RNAs are not produced directly in germ cells and that transposition into the germ cell genome requires ERV transmission from somatic cells. VLPs formed in the producing somatic cells could infect germ cells via the Env transmembrane protein, but other routes could also be used (Figure 1 panel 2). For instance, the *412* element can infect germ cells, although it does not encode Env [71]. This retrotransposon might use the Env protein encoded by another ERV for germ cell infection. Moreover, the *ZAM* ERV encodes an Env protein, but it reaches germ cells by usurping the endosome/exosome pathway in *Drosophila* ovaries for VLP transfer to the oocyte [59]. This route is normally employed for vitellogenin release and uptake by germ cells. It is not known whether *ZAM* also uses its Env protein to transit to germ cells. The detection of many new *ZAM* insertions in the progeny of flies in which *ZAM* is derepressed in somatic follicular cells indicates that after VLP transfer, *ZAM* can insert into the germ cells genome [72,73]. Therefore, this mechanism of propagation, using transfer from somatic to germ cells, is an efficient way to spread in a population. This suggests that ERVs expressed in somatic cells close to germ cells might create particles that infect the germline or might use a more passive mechanism to reach germ cells for insertion in the genome and vertical transmission.

It is clear that even if most ERVs are not expressed in the germline, they have many strategies for efficient propagation. Thanks to their capacity of intercellular and potentially inter-organism transfers, ERVs seem particularly well suited to efficiently propagate in an organism, to its descendants, and also to other species. This could explain why retrotransposons occupy such an important place in eukaryote genomes [8].

## 3. How to Deal with TE Invasion: Host Defense Strategies

### 3.1. Hybrid Dysgenesis and Discovery of the piRNA Pathway

HTT allows TEs to escape the host defenses through infection of a new species without a defense mechanism to block that specific TE. This situation can be compared to what happens when a pathogen enters an organism for the first time. The infected host must develop specific immune defenses for protection against the new invader. The organism response following HTT is largely unknown.

**Figure 2:**
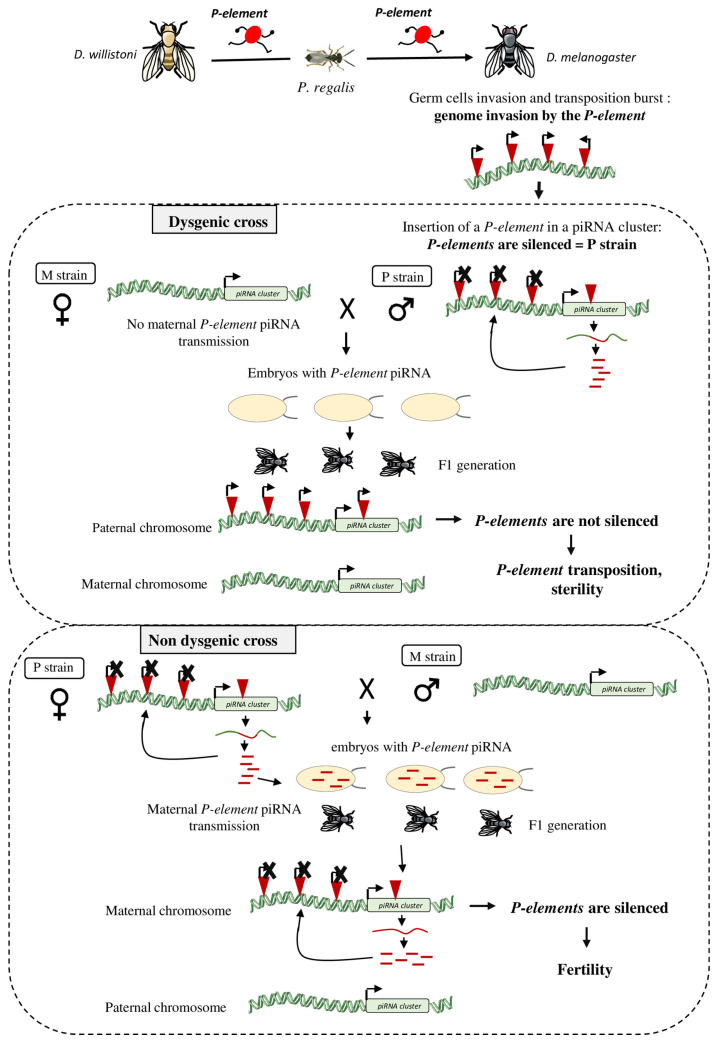
Host response following a TE horizontal transfer: example of the *D. melanogaster* genome invasion by the *P-element.* The *P-element* originally found in the *D. willistoni* genome has been transferred to *D. melanogaster* in the 1950s, possibly via the mite *Proctolaelaps regalis.* After germ cell invasion by the *P-element*, a transposition burst induced the creation of many *P-element* inheritable insertions. The *P-element* invaded the *D. melanogaster* genome and at one point it jumped into a piRNA cluster where piRNAs complementary to this element are produced to silence genomic *P-elements* (P strain). When a male with functional genomic *P-elements* (P strain) is crossed with a female without *P-element* (M strain), their offspring will be sterile (**dysgenic cross).** Indeed, the M female does not transfer any *P-element* piRNA to the embryos. These piRNAs are require to initiate the production of *P-element* piRNAs by the piRNA cluster present on the paternal chromosome of the offspring. Without *P-element* piRNA production in the F1 progeny, genomic *P-elements* are not silenced, leading to sterility. In the reverse cross (P female x M males), the F1 flies are fertile (**non-dysgenic cross).** Here, *P-element* piRNAs produced in the ovaries of the P mother are deposited in the embryos and allow the activation of the maternal *P-element*-containing piRNA cluster for the genomic *P-element* silencing.

The first data on the host response following HTT were obtained by studying *D. melanogaster* species invasion by the *P-element* (Figure 2). This HTT led to the invasion by *P-elements* of the genome of all wild-caught *D. melanogaster* between 1950 and 1980, demonstrating that at the time of invasion, before 1950, no defense mechanism provided efficient protection against *P-element* transposition. The invaded strains were called P strains and all strains isolated before the HTT (and thus without *P-elements* in their genome) were called M strains. Early studies showed that when females of M strains are crossed with males of P strains, the resulting F1 females are sterile and show rudimentary gonads, a phenomenon called hybrid dysgenesis [74,75]. Hybrid dysgenesis is also observed for other TEs, such as the *I-element* that invaded *D. melanogaster* from *Drosophila simulans* by HTT [76,77], Penelope [78], and *Hobo* [79]. Interestingly, hybrid dysgenesis is usually only observed at certain temperatures. Hybrid dysgenesis was the first evidence that differences in TE genomic content, due to HTT, can induce reproductive incompatibilities and might lead to speciation [80,81].

On the other hand, the reverse cross (P females x M males) leads to fertile F1 females and the *P-elements* are silenced in the F1 ovaries. This result revealed that some cytoplasmic components are maternally transmitted to the offspring and can trigger a TE-silencing response in the F1 gonads [82,83]. Later, the PIWI-interacting RNA (piRNAs) were discovered as the source of these cytoplasmic components [84,85].

#### 3.1.1. Discovery of the piRNA Pathway: A Barrier against Transposition

Many years after the first description of hybrid dysgenesis, several studies simultaneously reported the identification of a small RNA-based immune system, composed of PIWI-interacting RNA (piRNAs). This system, which was called the piRNA pathway [3,86,87,88,89,90], silences TEs genome-wide in male and female germ cells. piRNAs are small RNAs of 23–29 nucleotides in length that bind to PIWI proteins to silence TE activity via homology-dependent mechanisms. The components of this pathway are highly conserved: piRNAs and PIWI proteins are found in many organisms, from protozoans to higher eukaryotes [91,92]. This pathway has been extensively studied in *D. melanogaster* ovaries. These ovaries are formed by two cell types: follicular somatic cells and germ cells, and the piRNA pathway is active in both cell types. This model allowed showing that piRNAs are encoded by dedicated genomic loci called piRNA clusters [3]. piRNA clusters are composed of full length or truncated TEs that define the repertoire of elements recognized and silenced by the piRNA machinery. It is important to note that piRNA clusters have a tissue specific expression. In germ cells, piRNAs are loaded onto the PIWI proteins to trigger transcriptional silencing of TE [93]. Moreover, piRNAs loading onto the Aub and Ago3 proteins allows the amplification of the piRNA pool by a mechanism called the ping-pong cycle and post-transcriptional TE silencing [3,4]. Thus, in germ cells, TEs are silenced at both the transcriptional and post-transcriptional levels. In addition, Aub and Ago3 loaded with piRNAs produced during oogenesis are deposited in the early embryo and are then incorporated into the developing germ cells during embryogenesis [84,94,95]. This maternal deposition is required for efficient TE control in the offspring germline.

#### 3.1.2. Role of the piRNA Pathway in Hybrid Dysgenesis

The discovery of the piRNA pathway led to a better understanding of hybrid dysgenesis (Figure 2). Analysis of *P-element* piRNAs showed that they are produced in *D. melanogaster* lines invaded by *P-elements*, but not in lines without *P-elements*. During *P-element* invasion, the TE inserts at many loci, including in a piRNA-producing genomic region often located at the cytological site 1A [96,97]. Trapping *P-elements* into a piRNA cluster allows producing piRNAs complementary to this element and this can induce *P-element* silencing. Very often *P-elements* insert in the same piRNA cluster, specifically in telomere-associated sequences, located at the cytological site 1A [97,98,99,100]. *P-element* piRNAs produced in the ovaries of P strain females are then deposited in the embryo and are required to initiate the production of *P-element* piRNAs by a piRNA cluster in the offspring [84,101,102,103,104]. *P-element* piRNAs produced in F1 ovaries, together with PIWI proteins, could mediate transcriptional and post-transcriptional silencing of *P-element* euchromatic insertions. However, there is conflicting evidence about the exact effect of piRNA on *P-element* regulation [16]. Some studies have shown that *P-element* piRNAs could directly influence the expression of *P-elements* and thus modulate the level of *P-element* transcripts in germ cells [33,85]. On the other hand, one other study has demonstrated that *P-element* piRNAs act only on the splicing of P-element transcripts. *P-element* piRNAs promote the retention of the third intron and therefore inhibits the production of functional P-transposase in germ cells. [105]. Regardless of their mode of action, piRNAs are required to silence active *P-elements* in F1 gonads.

When no *P-element* piRNA is maternally deposited (M strain females), active *P-elements* inherited from the father are not silenced. Following *P-element* transposition, oogenesis is switched off and selective apoptosis is induced in ovarian stem cells by activation of the DNA damage checkpoint, leading to sterility (Figure 2). However, it should be noted that although males cannot transmit *P-element* piRNAs, the piRNA cluster containing the *P-element* is transmitted. During F1 hybrid dysgenic females culture at 25 °C, de novo production of *P-element* piRNAs is observed, possibly encoded by the paternally inherited *P-element*-containing piRNA cluster. *P-element* expression and transposition decrease and fertility is recovered [33,85]. This phenomenon shows the rapid adaptation to *P-element* expression in a single generation, with these flies inheriting a piRNA cluster for effective protection once activated.

Moreover, it has been shown that *Tirant* probably spread in *D. melanogaster* populations in ~1938, leaving behind old *Tirant* sequences in the heterochromatin [106,107]. These copies accumulated mutations and deletions, leading to highly degenerated *Tirant* sequences. These insertions produce piRNAs, but they are probably too degraded to induce an efficient silencing because a match with less than 10–20% of sequence divergence between piRNA and TE sequence seems to be required [108]. This means that new HTTs or TE reactivation might occur when the regulatory copies created by past invasions are too old and divergent relative to the corresponding active TEs.

Therefore, to be protected against an invading TE, organisms must put in place a new control system that will trigger TE silencing each time the memory copies become too divergent from the active copies.

### 3.2. Response to TE Invasion by Horizontal Transfer

Detection of the first individual of a species concerned by a HTT event is challenging because successful events are probably uncommon and unpredictable. Thus, the initial response of an organism following HTT is difficult to analyze. However, there are many cases where a TE has been transferred to a new species, and propagates in that population. This means that some individuals carry insertions of this new TE and others are devoid of it. The study of these ongoing HTT events allows monitoring the response at the population scale.

#### 3.2.1. Analysis of Ongoing TE Invasion

The *P-element* spread in *D. melanogaster* populations very rapidly because all wild-caught flies collected after 1980 carry *P-element* insertions. Since then, *P-element* propagation in *Drosophila* species continues, and 15 years ago the *P-element* began to invade *D. simulans* populations. Some *D. simulans* flies now have a huge number of *P-elements* in their genome (e.g., *D. simulans* collected in South Africa in 2012), others have only few insertions (*D. simulans* collected in Florida in 2010), and others do not harbor any *P-element* (*D. simulans* collected in the Sub-Saharan region in 2009). Before 1998, *P-elements* were completely absent in all *D. simulans* populations, suggesting an ongoing invasion process.

Kofler and al. studied the response to *P-element* invasion in a *D. simulans* population collected in 2010 and harboring few *P-element* insertions [38]. The copy number increase over generations suggested that *P-elements* are currently active in this population. However, after several generations (22 generations), *P-element* copy numbers in the populations stabilized. This observation was correlated with the new production of piRNAs complementary to *P-elements* and with the ping-pong signature. Based on their findings, the authors proposed a model called “shotgun silencing” composed of three different phases. First, the TE infects a new population where the piRNA pathway cannot silence *P-elements*, and the TE can transpose and multiply rapidly within the population. Second, during this wave of transposition, *P-element* inserted into a piRNA cluster in some individuals of the population resulting in the production of new piRNAs that are complementary to the *P-element*. However, *P-element* mobility is not controlled instantly at the population level, suggesting that these cluster insertions are not fixed in the population. In a third phase, *P-element* piRNA production increases in the population, possibly mediated by an increasing number of *P-element* insertions in piRNA clusters in several individuals, to a level sufficiently high to completely silence *P-elements* in the population. However, it is still unknown if one *P-element* insertion in a piRNA cluster is sufficient to repress this TE in one individual. Interestingly, it seems that complete silencing of the *P-element* in *D. melanogaster* can be mediated by only one insertion in a specific piRNA cluster at the 1A telomere-associated sequence locus [96,97].

Computer simulations of the dynamics of TE invasion have demonstrated that a single insertion in a single non-recombining cluster, such as the somatic piRNA cluster *flamenco*, is probably sufficient for TE repression [44]. Indeed, the *ZAM* retrotransposon is only present in one piRNA cluster, *flamenco*, and this single insertion is sufficient to produce enough *ZAM*-derived piRNAs to repress *ZAM* expression [109]. However, R. Kofler argues that, concerning piRNA clusters expressed in the germline, several cluster insertions are likely to be required to stop the invasion [44].

This genomic adaptation to invading TEs seems to be rapid and reproducible: most *D. simulans* individuals invaded by *P-elements* acquire a piRNA cluster insertion within a short period of time after invasion. However, the lag time between *P-element* invasion and silencing depends on the TE activity. *P-element* transposition seems to be temperature-dependent, and increases in flies raised at high temperature (≥23 °C) compared with flies raised at lower temperature (15 °C) where the P element transposase is less active [33,38,110]. High *P-element* activity results in many de novo insertions and increases the chances of insertion in piRNA clusters. Therefore, *P-element* repression is established faster when flies are raised at higher temperature. This example shows that adaptation to an invading TE can be rapid, but depends on the TE transposition activity.

#### 3.2.2. Analysis of the Initial Host Response to HTT

It is thought that *KoRV-A* is a recently introduced exogenous virus in many koala genomes and in the process of becoming an ERV. *KoRV*-A proviral insertions have been detected in almost all koala genomes analyzed, and there are now few naive populations [111]. *KoRV*-A is horizontally transmitted between animals and can infect the germline and integrate in the germ cell genome for vertical transmission [112,113,114]. Therefore, it is a great model to better understand how an organism and particularly how germ cells respond to retrovirus invasion. Yu et al. [115] suggested that after invasion by *KoRV*-A, an “innate” response is initiated in the gonads. This first phase is based on the recognition of a presumed conserved molecular pattern in *KoRV*-A RNA, leading to its processing into sense piRNA. *KoRV*-A RNAs are processed and are inefficient for active transposition. This finding shows that, during the initial response, insertion in a piRNA cluster is not necessary to trigger sense piRNAs production. This could be an efficient way to suppress virus replication in the first instance but does not appear to be sufficient to stably control KoRV-A in the population. The production of antisense piRNA is the second phase of the response and seems to require a TE insertion into a piRNA cluster. This will guide the sequence-specific adaptive immunity and generate a memory of the invader. Thus, the initial TE repression should not be too strong because the TE needs to be still sufficiently expressed to transpose and insert into a piRNA cluster. This study suggests that even when an innate mechanism allows the early repression of the TE, only insertion in a piRNA cluster leads to a robust, durable, and inheritable TE silencing.

Moreover, piRNAs could also be newly produced by euchromatic TE insertions converted into piRNA-producing loci, a phenomenon called paramutation [94,116,117,118]. Thus, numerous insertions dispersed across the genome may contribute to piRNA production and entire new piRNA generating regions could arise, providing another mechanism for acquiring immunity against new TEs. Indeed, spontaneous formation of novel piRNA clusters from euchromatic TE insertions and transgenic sequences has been observed [94,119,120]. At this time, it is still unknown how new piRNA producing genomic regions arise. It has been suggested that siRNAs could be involved in this process and thus participate in the initial detection of new elements and in the genesis of a stable piRNA response [119,121].

Interestingly, production of endogenous siRNAs (endo-siRNA) targeting TEs in somatic and germ cells had already been observed, suggesting a potential role of these small RNAs in the initial phase of TE control [122,123,124,125]. As an example, Rozhkov et al. developed an artificial system to follow the initial response following TE invasion and have also detected a rapid production of siRNA [126]. The authors introduced a transgene containing an active copy of *Penelope* in the *D. melanogaster* genome to mimic an invasion by a new TE. *Penelope* is a TE found in *Drosophila virilis,* but absent in *D. melanogaster*. After this artificial HT, they observed production of siRNAs against *Penelope* in the transgenic flies. Endo-siRNAs are known to be produced from a dsRNA precursor cleaved by Dicer-2. This precursor does not seem to predominantly derive from master loci, as inferred for TE piRNAs produced by piRNA clusters [123]. The dsRNA might rather be produced by the pairing of sense and antisense transcripts generated by bidirectional transcription of TEs or by the association of transposon sense RNA with antisense RNA transcribed from a piRNA cluster. Moreover, a mutation in the coding gene of Dicer2 or Ago2, two proteins required for siRNA production and targeting, triggers derepression of some TEs in *Drosophila* S2 cells and in *Drosophila* ovaries [122,123,127,128]. However, in the case of the artificial HTT, at the time of the TE introduction in the *D. melanogaster* genome, *Penelope* was not inserted at any loci in the genome. Therefore, this new siRNA production probably comes from the processing of newly produced retrotransposon RNAs. It might be a kind of first “immune defense” initiated by the arrival of an invader in the cell, as observed for viruses. However, siRNA production was not sufficient to silence completely *Penelope* in *D. melanogaster* and it could still transpose occasionally. At later stages of genome invasion, *Penelope*-derived piRNA production was observed and this correlated with the presence of a new *Penelope* insertion in a piRNA cluster [129]. A number of transposition events might be required for insertion in a piRNA cluster and to set up an efficient control of the invading TE via the production of related piRNAs. Moreover, the cooperation between siRNA and piRNA production in the fight against *Penelope* has not been studied yet. It seems that siRNA production precedes or accompanies piRNA production and could then be potentially involved in the activation of piRNA production [119,121]. However, these findings support the idea that the piRNA response is a robust response that must be firmly established to efficiently control the invading TE.

That being said, it is also interesting to note that, while piRNAs seem to be absent in most of the nematode species [130], some evidence suggests that horizontally transferred TEs within these species are controlled very rapidly after their entrance in new genomes. Single-copy horizontally transferred DNA transposons, almost intact copies, have been found in nematode genomes [24]. Thus, it is possible that another efficient mechanism could control the transposition just after the HTT, independently of the production of piRNA.

### 3.3. Germ Cell Response to TE Invasion from Neighboring Somatic Cells

#### 3.3.1. piRNA Clusters Keep the Memory of Past Invasions to Protect Germ Cells

Thanks to piRNAs produced by piRNA clusters, TEs are controlled and this immunity is transmitted to the next generation. Interestingly, a recent study by Gebert et al. has challenged this assumption [116]. The authors have demonstrated that deletion of the three largest piRNA clusters expressed in Drosophila germ cells has no effect on TE regulation or fertility. Although much redundancy between multiple germline piRNA clusters is observed for many TEs, this study proposes that, in germ cells, piRNA produced by dispersed elements could be sufficient for TE regulation. However, divergent observations have been made analyzing the *flamenco* piRNA cluster, which is only expressed in the ovary somatic cells [3,67]. A study has been undertaken on a *Drosophila* line called RevI-H2 [73] where the *ZAM* retrotransposon was deleted from its regulatory piRNA cluster *flamenco*. In wild type flies, *ZAM* is present in *flamenco,* and 85% of all *ZAM*-derived piRNAs are produced by the *flamenco* piRNA cluster. *Flamenco* is only expressed in the ovary somatic cells and no *ZAM* insertion is detected in any other piRNA clusters in the reference genome. This means that *flamenco* is the only regulatory piRNA cluster of *ZAM*. It is important to note that *ZAM* deletion from the *flamenco* piRNA cluster results in loss of *ZAM* control [72,73,109,131], demonstrating the correlation between the presence of a TE in a somatic piRNA cluster and the regulation of this TE. In RevIH2 ovaries, *ZAM* is expressed in follicular cells and forms pseudo-viral particles (VLPs) that are assumed to use the endosomal vitellogenin trafficking system to enter the oocyte and invade the germline [59]. *ZAM* deletion from its regulatory piRNA cluster in somatic follicular cells leads to germ cell invasion by *ZAM* VLPs. This condition can be compared to what happens when a TE first invades a new species through horizontal transfer and reaches the germ cells. Therefore, the study of what happens in the germline after *ZAM* invasion can bring new insights into how germ cells control TE propagation. Indeed, the RevI-H2 line adapted to *ZAM* invasion by trapping a new *ZAM* copy in a germline piRNA cluster. This led to the production of *ZAM*-derived piRNAs in the germline [72]. This could result from the specific targeting of *ZAM* to piRNA clusters or from a random transposition event that has been selected through time. Lastly, the time required to trap a TE in a regulatory piRNA cluster is not known, and TE silencing establishment might take several generations.

#### 3.3.2. How Long Does It Take to Implement an Efficient Response against an Invading TE?

All these studies revealed that trapping in a piRNA cluster is an efficient process to control transposition of an invading TE and to protect the genome. However, the time required for such an event remains unknown. Depletion in somatic *Drosophila* cells of PIWI, a protein required for TE silencing, leads to derepression of many TEs, including several retrotransposons that may invade germ cells [71]. In this study, new individual TE insertions were detected during more than 73 generations, but did not become fixed in the population. These TE insertions are not particularly enriched at piRNA clusters and another study revealed that the number of TE cluster insertions is insufficient with expectations of the “Trap model” that proposes a specific trapping of TEs in piRNA clusters [38,132]. Moreover, several piRNA cluster insertions per individual seem to be necessary to stop the invasion [44]. All these different arguments might explain why it takes several generations to establish a control. Furthermore, how such control is put in place may vary for each TE and each invasion event. Several parameters should be considered: (i) TE invasions rely on many processes (e.g., cell transfer, pseudo-viral particle formation, vector use) and the response might be different for each mechanism; (ii) TEs have different transposition mechanisms and spatiotemporal expression, resulting in variable transposition rates. A high transposition rate may increase the chances of successful insertion into a piRNA cluster without any specific trapping at these loci; (iii) environmental conditions (e.g., temperature) also influence the transposition rate [33] and modify the response following invasion; (iv) piRNA production varies among all piRNA clusters found in genomes. Moreover, piRNA clusters show a tissue-specific expression and are not all expressed in germ cells where transposition needs to be silenced. This highlights that individual TE insertions into piRNA clusters can have different effects on TE taming.

Thus, although TE trapping in piRNA clusters seems to be an efficient way to sustainably control TE transposition, the response to each TE invasion will be specific. A detailed analysis of many different invasion processes is necessary to better understand the parameters influencing the establishment of the silencing response. Currently, it is still unknown whether TEs insert preferentially into piRNA clusters, or whether they insert randomly in the genome and then insertions in piRNA clusters are retained during evolution due to their beneficial effects. Moreover, TEs may insert, but can also be deleted from a piRNA cluster. Indeed, piRNA clusters in flies and mammals evolve rapidly [85,116,133,134]. Consequently, the piRNA repertoire produced by germ cells evolves constantly and can determine, at a specific moment, TE activity and invasion. Importantly, piRNA clusters keep a memory of past invasions to protect the genome from active and threatening TEs. This strategy can be compared to an adaptive immune system that adjusts to each TE’s characteristics and activity.

## 4. Conclusions

TEs and host control mechanisms evolve constantly to find a balance between transposition and repression. It is now acknowledged that genomes have coevolved with their TEs, developing strategies to limit transposition. On the other side, TEs propagate in genomes by HTT that allows crossing species boundaries and by targeting germ cells for transposition to ensure their transmission to the next generation. It seems clear that organisms also have evolutionary advantages by allowing some transposition because TEs can be seen as a trigger of a series of events that actively shape the genome architecture and give rise to biological innovations. However, transposition is also a threatening phenomenon because it may create deleterious mutations that could endanger the species’ survival. This can explain why organisms develop sophisticated and adaptive strategies to control TE transposition and propagation. Although the initial response following HTT is still largely unknown, it appears that TE insertion in a piRNA cluster is a very efficient mechanism for the long-term control of TE transposition. Studying all TE copies and TE remnants in genomes can provide information about the “history” of each genome facing TE invasion. Each species and each individual has a very specific TE insertion profile, indicating that each organism must deal with specific transposition phenomena and must adapt individually. This is a beautiful example of genome adaptation and demonstrates that a potential source of danger can also be beneficial if well controlled, turning from “junk” into “gold”.

## Data Availability

Not applicable.

## References

[1] McClintock B. (1953). Induction of Instability at Selected Loci in Maize. Genetics.

[2] Wicker T., Sabot F., Hua-Van A., Bennetzen J.L., Capy P., Chalhoub B., Flavell A., Leroy P., Morgante M., Panaud O. (2007). A unified classification system for eukaryotic transposable elements. Nat. Rev. Genet..

[3] Brennecke J., Aravin A.A., Stark A., Dus M., Kellis M., Sachidanandam R., Hannon G.J. (2007). Discrete small RNA-generating loci as master regulators of transposon activity in Drosophila. Cell.

[4] Gunawardane L.S., Saito K., Nishida K.M., Miyoshi K., Kawamura Y., Nagami T., Siomi H., Siomi M.C. (2007). A slicer-mediated mechanism for repeat-associated siRNA 5’ end formation in Drosophila. Science.

[5] Slotkin R.K., Vaughn M., Borges F., Tanurdžić M., Becker J.D., Feijó J.A., Martienssen R.A. (2009). Epigenetic Reprogramming and Small RNA Silencing of Transposable Elements in Pollen. Cell.

[6] Wolf D., Goff S.P. (2009). Embryonic stem cells use ZFP809 to silence retroviral DNAs. Nature.

[7] Wolf G., Yang P., Füchtbauer A.C., Füchtbauer E.-M., Silva A.M., Park C., Wu W., Nielsen A.L., Pedersen F.S., Macfarlan T.S. (2015). The KRAB zinc finger protein ZFP809 is required to initiate epigenetic silencing of endogenous retroviruses. Genes Dev..

[8] Huang C.R.L., Burns K.H., Boeke J.D. (2012). Active Transposition in Genomes. Annu. Rev. Genet..

[9] Zeh D.W., Zeh J.A., Ishida Y. (2009). Transposable elements and an epigenetic basis for punctuated equilibria. BioEssays.

[10] Cordaux R., Batzer M.A. (2009). The impact of retrotransposons on human genome evolution. Nat. Rev. Genet..

[11] Feschotte C., Pritham E.J. (2007). DNA transposons and the evolution of eukaryotic genomes. Annu. Rev. Genet..

[12] Oliver K.R., Greene W.K. (2009). Transposable elements: Powerful facilitators of evolution. BioEssays.

[13] Daniels S.B., Peterson K.R., Strausbaugh L.D., Kidwell M.G., Chovnik A. (1990). Evidence for horizontal transmission of the P transposable element between Drosophila species. Genetics.

[14] Anxolabéhère D., Nouaud D., Périquet G., Tchen P. (1985). P-element distribution in Eurasian populations of Drosophila melanogaster: A genetic and molecular analysis. Proc. Natl. Acad. Sci. USA.

[15] Anxolabéhère D., Kidwell M.G., Periquet G. (1988). Molecular characteristics of diverse populations are consistent with the hypothesis of a recent invasion of Drosophila melanogaster by mobile P elements. Mol. Biol. Evol..

[16] Ghanim G.E., Rio D.C., Teixeira F.K. (2020). Mechanism and regulation of P element transposition. Open Biol..

[17] Peccoud J., Loiseau V., Cordaux R., Gilbert C. (2017). Massive horizontal transfer of transposable elements in insects. Proc. Natl. Acad. Sci. USA.

[18] Houck M.A., Clark J.B., Peterson K.R., Kidwell M.G. (1991). Possible horizontal transfer of Drosophila genes by the mite Proctolaelaps regalis. Science.

[19] Loreto E.L.S., Carareto C.M.A., Capy P. (2008). Revisiting horizontal transfer of transposable elements in Drosophila. Heredity.

[20] Gilbert C., Peccoud J., Chateigner A., Moumen B., Cordaux R., Herniou E.A. (2016). Continuous Influx of Genetic Material from Host to Virus Populations. PLoS Genet..

[21] Piskurek O., Okada N. (2007). Poxviruses as possible vectors for horizontal transfer of retroposons from reptiles to mammals. Proc. Natl. Acad. Sci. USA.

[22] Dunning Hotopp J.C., Clark M.E., Oliveira D.C.S.G., Foster J.M., Fischer P., Muñoz Torres M.C., Giebel J.D., Kumar N., Ishmael N., Wang S. (2007). Widespread lateral gene transfer from intracellular bacteria to multicellular eukaryotes. Science.

[23] Raychoudhury R., Baldo L., Oliveira D.C.S.G., Werren J.H. (2009). Modes of acquisition of Wolbachia: Horizontal transfer, hybrid introgression, and codivergence in the Nasonia species complex. Evol. Int. J. Org. Evol..

[24] Palazzo A., Escuder E., D’Addabbo P., Lovero D., Marsano R.M. (2021). A genomic survey of Tc1-mariner transposons in nematodes suggests extensive horizontal transposon transfer events. Mol. Phylogenet. Evol..

[25] White P.M., Pietri J.E., Debec A., Russell S., Patel B., Sullivan W. (2017). Mechanisms of Horizontal Cell-to-Cell Tranfer of Wolbachia spp. in Drosophila melanogaster. Appl. Environ. Microbiol..

[26] Stroun M., Lyautey J., Lederrey C., Mulcahy H.E., Anker P. (2001). Alu repeat sequences are present in increased proportions compared to a unique gene in plasma/serum DNA: Evidence for a preferential release from viable cells?. Ann. N. Y. Acad. Sci..

[27] Kawamura Y., Sanchez Calle A., Yamamoto Y., Sato T.A., Ochiya T. (2019). Extracellular vesicles mediate the horizontal transfer of an active LINE-1 retrotransposon. J. Extracell. Vesicles.

[28] Tkach M., Théry C. (2016). Communication by Extracellular Vesicles: Where We Are and Where We Need to Go. Cell.

[29] Wang L., Dou K., Moon S., Tan F.J., Zhang Z.Z. (2018). Hijacking Oogenesis Enables Massive Propagation of LINE and Retroviral Transposons. Cell.

[30] Tiwari B., Kurtz P., Jones A.E., Wylie A., Amatruda J.F., Boggupalli D.P., Gonsalvez G.B., Abrams J.M. (2017). Retrotransposons Mimic Germ Plasm Determinants to Promote Transgenerational Inheritance. Curr. Biol..

[31] Shpiz S., Kwon D., Uneva A., Kim M., Klenov M., Rozovsky Y., Georgiev P., Savitsky M., Kalmykova A. (2007). Characterization of Drosophila telomeric retroelement TAHRE: Transcription, transpositions, and RNAi-based regulation of expression. Mol. Biol. Evol..

[32] Van De Bor V., Hartswood E., Jones C., Finnegan D., Davis I. (2005). gurken and the I factor retrotransposon RNAs share common localization signals and machinery. Dev. Cell.

[33] Moon S., Cassani M., Lin Y.A., Wang L., Dou K., Zhang Z.Z. (2018). A Robust Transposon-Endogenizing Response from Germline Stem Cells. Dev. Cell.

[34] Suh D.S., Choi E.H., Yamazaki T., Harada K. (1995). Studies on the transposition rates of mobile genetic elements in a natural population of Drosophila melanogaster. Mol. Biol. Evol..

[35] Nuzhdin S.V., Mackay T.F. (1995). The genomic rate of transposable element movement in Drosophila melanogaster. Mol. Biol. Evol..

[36] Maside X., Assimacopoulos S., Charlesworth B. (2000). Rates of movement of transposable elements on the second chromosome of Drosophila melanogaster. Genet. Res..

[37] Le Rouzic A., Capy P. (2005). The first steps of transposable elements invasion: Parasitic strategy vs. genetic drift. Genetics.

[38] Kofler R., Senti K.A., Nolte V., Tobler R., Schlötterer C. (2018). Molecular dissection of a natural transposable element invasion. Genome Res..

[39] Robillard É., Le Rouzic A., Zhang Z., Capy P., Hua-Van A. (2016). Experimental evolution reveals hyperparasitic interactions among transposable elements. Proc. Natl. Acad. Sci. USA.

[40] Schaack S., Gilbert C., Feschotte C. (2010). Promiscuous DNA: Horizontal transfer of transposable elements and why it matters for eukaryotic evolution. Trends Ecol. Evol..

[41] Zhang H.H., Peccoud J., Xu M.R.X., Zhang X.G., Gilbert C. (2020). Horizontal transfer and evolution of transposable elements in vertebrates. Nat. Commun..

[42] Capy P. (2021). Taming, Domestication and Exaptation: Trajectories of Transposable Elements in Genomes. Cells.

[43] Hickey D.A. (1982). Selfish DNA: A sexually transmitted nuclear parasite. Genetics.

[44] Kofler R. (2019). Dynamics of transposable element invasions with piRNA clusters. Mol. Biol. Evol..

[45] Laski F.A., Rio D.C., Rubin G.M. (1986). Tissue specificity of Drosophila P element transposition is regulated at the level of mRNA splicing. Cell.

[46] Chaboissier M.C., Busseau I., Prosser J., Finnegan D.J., Bucheton A. (1990). Identification of a potential RNA intermediate for transposition of the LINE-like element I factor in Drosophila melanogaster. EMBO J..

[47] del Carmen Seleme M., Disson O., Robin S., Brun C., Teninges D., Bucheton A. (2005). In vivo RNA localization of I factor, a non-LTR retrotransposon, requires a cis-acting signal in ORF2 and ORF1 protein. Nucleic Acids Res..

[48] Rogers R.L., Slatkin M. (2017). Excess of genomic defects in a woolly mammoth on Wrangel island. PLoS Genet..

[49] Platt R.N., Ray D.A. (2012). A non-LTR retroelement extinction in Spermophilus tridecemlineatus. Gene.

[50] Dufourt J., Vaury C. (2014). During a short window of Drosophila oogenesis, piRNA biogenesis may be boosted and mobilization of transposable elements allowed. Front. Genet..

[51] Théron E., Maupetit-Mehouas S., Pouchin P., Baudet L., Brasset E., Vaury C. (2018). The interplay between the argonaute proteins piwi and aub within drosophila germarium is critical for oogenesis, piRNA biogenesis and TE silencing. Nucleic Acids Res..

[52] Dufourt J., Dennis C., Boivin A., Gueguen N., Théron E., Goriaux C., Pouchin P., Ronsseray S., Brasset E., Vaury C. (2014). Spatio-temporal requirements for transposable element piRNA-mediated silencing during Drosophila oogenesis. Nucleic Acids Res..

[53] Seisenberger S., Andrews S., Krueger F., Arand J., Walter J., Santos F., Popp C., Thienpont B., Dean W., Reik W. (2012). The Dynamics of Genome-wide DNA Methylation Reprogramming in Mouse Primordial Germ Cells. Mol. Cell.

[54] Evsikov A.V., De Vries W.N., Peaston A.E., Radford E.E., Fancher K.S., Chen F.H., Blake J.A., Bult C.J., Latham K.E., Solter D. (2004). Systems biology of the 2-cell mouse embryo. Cytogenet. Genome Res..

[55] Fadloun A., Le Gras S., Jost B., Ziegler-Birling C., Takahashi H., Gorab E., Carninci P., Torres-Padilla M.E. (2013). Chromatin signatures and retrotransposon profiling in mouse embryos reveal regulation of LINE-1 by RNA. Nat. Struct. Mol. Biol..

[56] Kigami D., Minami N., Takayama H., Imai H. (2003). MuERV-L is one of the earliest transcribed genes in mouse one-cell embryos. Biol. Reprod..

[57] Maupetit-Mehouas S., Vaury C. (2020). Transposon Reactivation in the Germline May Be Useful for Both Transposons and Their Host Genomes. Cells.

[58] Chalvet F., Teysset L., Terzian C., Prud’homme N., Santamaria P., Bucheton A., Pélisson A. (1999). Proviral amplification of the Gypsy endogenous retrovirus of Drosophila melanogaster involves env-independent invasion of the female germline. EMBO J..

[59] Brasset E., Taddei A.R., Arnaud F., Faye B., Fausto A.M., Mazzini M., Giorgi F., Vaury C. (2006). Viral particles of the endogenous retrovirus ZAM from Drosophila melanogaster use a pre-existing endosome/exosome pathway for transfer to the oocyte. Retrovirology.

[60] Syomin B.V., Fedorova L.I., Surkov S.A., Ilyin Y.V. (2001). The endogenous Drosophila melanogaster retrovirus gypsy can propagate in Drosophila hydei cells. Mol. Gen. Genet..

[61] Keegan R.M., Talbot L.R., Chang Y.H., Metzger M.J., Dubnau J. (2021). Intercellular viral spread and intracellular transposition of Drosophila gypsy. PLoS Genet..

[62] Kim A., Terzian C., Santamaria P., Pélisson A., Prud’homme N., Bucheton A. (1994). Retroviruses in invertebrates: The gypsy retrotransposon is apparently an infectious retrovirus of Drosophila melanogaster. Proc. Natl. Acad. Sci. USA.

[63] Leblanc P., Desset S., Giorgi F., Taddei A.R., Fausto A.M., Mazzini M., Dastugue B., Vaury C. (2002). Life Cycle of an Endogenous Retrovirus, ZAM, in Drosophila melanogaster. J. Virol..

[64] Tcheressiz S., Calco V., Arnaud F., Arthaud L., Dastugue B., Vaury C. (2002). Expression of the Idefix retrotransposon in early follicle cells in the germarium of Drosophila melanogaster is determined by its LTR sequences and a specific genomic context. Mol. Genet. Genomics.

[65] Malone C.D., Brennecke J., Dus M., Stark A., McCombie W.R., Sachidanandam R., Hannon G.J. (2009). Specialized piRNA Pathways Act in Germline and Somatic Tissues of the Drosophila Ovary. Cell.

[66] Olivieri D., Sykora M.M., Sachidanandam R., Mechtler K., Brennecke J. (2010). An in vivo RNAi assay identifies major genetic and cellular requirements for primary piRNA biogenesis in Drosophila. EMBO J..

[67] Pélisson A., Song S.U., Prud’homme N., Smith P.A., Bucheton A., Corces V.G. (1994). Gypsy transposition correlates with the production of a retroviral envelope-like protein under the tissue-specific control of the Drosophila flamenco gene. EMBO J..

[68] Sokolova O.A., Mikhaleva E.A., Kharitonov S.L., Abramov Y.A., Gvozdev V.A., Klenov M.S. (2020). Special vulnerability of somatic niche cells to transposable element activation in Drosophila larval ovaries. Sci. Rep..

[69] Cavarec L., Jensen S., Casella J.F., Cristescu S.A., Heidmann T. (1997). Molecular cloning and characterization of a transcription factor for the copia retrotransposon with homology to the BTB-containing lola neurogenic factor. Mol. Cell. Biol..

[70] Meignin C., Dastugue B., Vaury C. (2004). Intercellular communication between germ line and somatic line is utilized to control the transcription of ZAM, an endogenous retrovirus from Drosophila melanogaster. Nucleic Acids Res..

[71] Barckmann B., El-Barouk M., Pélisson A., Mugat B., Li B., Franckhauser C., Fiston Lavier A.-S., Mirouze M., Fablet M., Chambeyron S. (2018). The somatic piRNA pathway controls germline transposition over generations. Nucleic Acids Res..

[72] Duc C., Yoth M., Jensen S., Mouniée N., Bergman C.M., Vaury C., Brasset E. (2019). Trapping a somatic endogenous retrovirus into a germline piRNA cluster immunizes the germline against further invasion. Genome Biol..

[73] Desset S., Conte C., Dimitri P., Calco V., Dastugue B., Vaury C. (1999). Mobilization of two retroelements, ZAM and Idefix, in a novel unstable line of Drosophila melanogaster. Mol. Biol. Evol..

[74] Kidwell M.G., Kidwell J.F., Sved J.A. (1977). Hybrid dysgenesis in Drosophila melanogaster: A syndrome of aberrant traits including mutation, sterility and male recombination. Genetics.

[75] Rubin G.M., Kidwell M.G., Bingham P.M. (1982). The molecular basis of P-M hybrid dysgenesis: The nature of induced mutations. Cell.

[76] Picard G. (1976). Non mendelian female sterility in Drosophila melanogaster: Hereditary transmission of I factor. Genetics.

[77] Busseau I., Chaboissier M.C., Pélisson A., Bucheton A. (1994). I factors in Drosophila melanogaster: Transposition under control. Genetica.

[78] Evgen’ev M.B., Zelentsova H., Shostak N., Kozitsina M., Barskyi V., Lankenau D.H., Corces V.G. (1997). Penelope, a new family of transposable elements and its possible role in hybrid dysgenesis in Drosophila virilis. Proc. Natl. Acad. Sci. USA.

[79] Blackman R.K., Grimaila R., Macy M., Koehler D., Gelbart W.M. (1987). Mobilization of hobo elements residing within the decapentaplegic gene complex: Suggestion of a new hybrid dysgenesis system in Drosophila melanogaster. Cell.

[80] Serrato-Capuchina A., Matute D.R. (2018). The role of transposable elements in speciation. Genes.

[81] Belyayev A. (2014). Bursts of transposable elements as an evolutionary driving force. J. Evol. Biol..

[82] Engels W.R. (2007). Hybrid dysgenesis in Drosophila melanogaster: Rules of inheritance of female sterility. Genet. Res..

[83] Kidwell M.G. (1983). Hybrid dysgenesis in Drosophila melanogaster: Factors affecting chromosomal contamination in the P-M system. Genetics.

[84] Brennecke J., Malone C.D., Aravin A.A., Sachidanandam R., Stark A., Hannon G.J. (2008). An Epigenetic Role for Maternally Inherited piRNAs in Transposon Silencing. Science.

[85] Khurana J.S., Wang J., Xu J., Koppetsch B.S., Thomson T.C., Nowosielska A., Li C., Zamore P.D., Weng Z., Theurkauf W.E. (2011). Adaptation to P element transposon invasion in drosophila melanogaster. Cell.

[86] Aravin A., Gaidatzis D., Iovino N., Morris P., Brownstein M.J., Kuramochi-miyagawa S., Nakano T., Chien M., Russo J.J., Ju J. (2006). A novel class of small RNAs bind to MILI protein in mouse testes. Nature.

[87] Girard A., Sachidanandam R., Hannon G.J., Carmell M.A. (2006). A germline-specific class of small RNAs binds mammalian Piwi proteins. Nature.

[88] Grivna S.T., Beyret E., Wang Z., Lin H. (2006). A novel class of small RNAs in mouse spermatogenic cells. Genes Dev..

[89] Lau N.C., Seto A.G., Kim J., Kuramochi-Miyagawa S., Nakano T., Bartel D.P., Kingston R.E. (2006). Characterization of the piRNA complex from rat testes. Science.

[90] Watanabe T., Takeda A., Tsukiyama T., Mise K., Okuno T., Sasaki H., Minami N., Imai H. (2006). Identification and characterization of two novel classes of small RNAs in the mouse germline: Retrotransposon-derived siRNAs in oocytes and germline small RNAs in testes. Genes Dev..

[91] Gainetdinov I., Colpan C., Arif A., Cecchini K., Zamore P.D. (2018). A Single Mechanism of Biogenesis, Initiated and Directed by PIWI Proteins, Explains piRNA Production in Most Animals. Mol. Cell.

[92] Bhattacharya S., Bakre A., Bhattacharya A. (2002). Mobile genetic elements in protozoan parasites. J. Genet..

[93] Sienski G., Dönertas D., Brennecke J. (2012). Transcriptional silencing of transposons by Piwi and maelstrom and its impact on chromatin state and gene expression. Cell.

[94] De Vanssay A., Bougé A.L., Boivin A., Hermant C., Teysset L., Delmarre V., Antoniewski C., Ronsseray S. (2012). Paramutation in Drosophila linked to emergence of a piRNA-producing locus. Nature.

[95] Le Thomas A., Stuwe E., Li S., Du J., Marinov G., Rozhkov N., Chen Y.C.A., Luo Y., Sachidanandam R., Toth K.F. (2014). Transgenerationally inherited piRNAs trigger piRNA biogenesis by changing the chromatin of piRNA clusters and inducing precursor processing. Genes Dev..

[96] Marin L., Lehmann M., Nouaud D., Izaabel H., Anxolabéhère D., Ronsseray S. (2000). P-element repression in Drosophila melanogaster by a naturally occurring defective telomeric P copy. Genetics.

[97] Ronsseray S., Lehmann M., Anxolabehere D. (1991). The maternally inherited regulation of P elements in Drosophila melanogaster can be elicited by two P copies at cytological site 1A on the X chromosome. Genetics.

[98] Ronsseray S., Josse T., Boivin A., Anxolabéhère D. (2003). Telomeric transgenes and trans-silencing in Drosophila. Genetica.

[99] Casier K., Delmarre V., Gueguen N., Hermant C., Viodé E., Vaury C., Ronsseray S., Brasset E., Teysset L., Boivin A. (2019). Environmentally-induced epigenetic conversion of a piRNA cluster. Elife.

[100] Marie P.P., Ronsseray S., Boivin A. (2017). From embryo to adult: PiRNA-mediated silencing throughout germline development in *Drosophila*. G3 Genes Genomes Genet..

[101] Hermant C., Boivin A., Teysset L., Delmarre V., Asif-Laidin A., Van Den Beek M., Antoniewski C., Ronsseray S. (2015). Paramutation in drosophila requires both nuclear and cytoplasmic actors of the piRNA pathway and induces cis-spreading of piRNA production. Genetics.

[102] de Vanssay A., Bougé A.L., Boivin A., Hermant C., Teysset L., Delmarre V., Antoniewski C., Ronsseray S. (2013). piRNAs and epigenetic conversion in Drosophila. Fly.

[103] Todeschini A.L., Teysset L., Delmarre V., Ronsseray S. (2010). The epigenetic Trans-silencing effect in Drosophila involves maternally-transmitted small RNAs whose production depends on the piRNA pathway and HP1. PLoS ONE.

[104] Josse T., Teysset L., Todeschini A.L., Sidor C.M., Anxolabéhère D., Ronsseray S. (2007). Telomeric trans-silencing: An epigenetic repression combining RNA silencing and heterochromatin formation. PLoS Genet..

[105] Teixeira F.K., Okuniewska M., Malone C.D., Coux R.X., Rio D.C., Lehmann R. (2017). PiRNA-mediated regulation of transposon alternative splicing in the soma and germ line. Nature.

[106] Mugnier N., Gueguen L., Vieira C., Biémont C. (2008). The heterochromatic copies of the LTR retrotransposons as a record of the genomic events that have shaped the Drosophila melanogaster genome. Gene.

[107] Moltó M.D., Paricio N., López-Preciado M.A., Semeshin V.F., Martínez-Sebastián M.J. (1996). Tirant: A new retrotransposon-like element in Drosophila melanogaster. J. Mol. Evol..

[108] Schwarz F., Wierzbicki F., Senti K.-A., Kofler R. (2021). Tirant stealthily invaded natural Drosophila melanogaster populations during the last century. Mol. Biol. Evol..

[109] Zanni V., Eymery A., Coiffet M., Zytnicki M., Luyten I., Quesneville H., Vaury C., Jensen S. (2013). Distribution, evolution, and diversity of retrotransposons at the flamenco locus reflect the regulatory properties of piRNA clusters. Proc. Natl. Acad. Sci. USA.

[110] Schaefer R.E., Kidwell Z.M.G., Fausto-sterling A. (1979). Hybrid dysgenesis in drosophila melanogaster: Morphological and cytological studies of ovarian dysgenesis. Public Health.

[111] Tarlinton R.E., Meers J., Young P.R. (2006). Retroviral invasion of the koala genome. Nature.

[112] Chappell K.J., Brealey J.C., Amarilla A.A., Watterson D., Hulse L., Palmieri C., Johnston S.D., Holmes E.C., Meers J., Young P.R. (2017). Phylogenetic Diversity of Koala Retrovirus within a Wild Koala Population. J. Virol..

[113] Denner J., Young P.R. (2013). Koala retroviruses: Characterization and impact on the life of koalas. Retrovirology.

[114] Ishida Y., Zhao K., Greenwood A.D., Roca A.L. (2015). Proliferation of endogenous retroviruses in the early stages of a host germ line invasion. Mol. Biol. Evol..

[115] Yu T., Koppetsch B., Chappell K., Pagliarani S., Johnston S., Silverstein N.J., Luban J., Weng Z., Thauerkauf W.E. (2019). The PiRNA Response to Retroviral Invasion of the Koala Genome. Cell.

[116] Gebert D., Neubert L.K., Lloyd C., Gui J., Lehmann R., Teixeira F.K. (2021). Large Drosophila germline piRNA clusters are evolutionarily labile and dispensable for transposon regulation. Mol. Cell.

[117] Andersen P.R., Tirian L., Vunjak M., Brennecke J. (2017). A heterochromatin-dependent transcription machinery drives piRNA expression. Nature.

[118] Mohn F., Sienski G., Handler D., Brennecke J. (2014). The Rhino-Deadlock-Cutoff complex licenses noncanonical transcription of dual-strand piRNA clusters in Drosophila. Cell.

[119] Olovnikov I.A., Kalmykova A.I. (2013). piRNA clusters as a main source of small RNAs in the animal germline. Biochemistry.

[120] Shpiz S., Ryazansky S., Olovnikov I., Abramov Y., Kalmykova A. (2014). Euchromatic Transposon Insertions Trigger Production of Novel Pi- and Endo-siRNAs at the Target Sites in the Drosophila Germline. PLoS Genet..

[121] Luo Y., He P., Kanrar N., Toth K.F., Aravin A.A. (2022). Maternally inherited siRNAs initiate piRNA cluster formation. bioRxiv.

[122] Czech B., Malone C.D., Zhou R., Stark A., Schlingeheyde C., Dus M., Perrimon N., Kellis M., Wohlschlegel J.A., Sachidanandam R. (2008). An endogenous small interfering RNA pathway in Drosophila. Nature.

[123] Chung W.J., Okamura K., Martin R., Lai E.C. (2008). Endogenous RNA Interference Provides a Somatic Defense against Drosophila Transposons. Curr. Biol..

[124] Ghildiyal M., Zamore P.D. (2009). Small silencing RNAs: An expanding universe. Nat. Rev. Genet..

[125] Okamura K., Chung W.J., Ruby J.G., Guo H., Bartel D.P., Lai E.C. (2008). The Drosophila hairpin RNA pathway generates endogenous short interfering RNAs. Nature.

[126] Rozhkov N.V., Aravin A.A., Zelentsova E.S., Schostak N.G., Sachidanandam R., Mccombie W.R., Hannon G.J., Evgen’ev M.B. (2010). Small RNA-based silencing strategies for transposons in the process of invading Drosophila species. RNA.

[127] Rehwinkel J., Natalin P., Stark A., Brennecke J., Cohen S.M., Izaurralde E. (2006). Genome-Wide Analysis of mRNAs Regulated by Drosha and Argonaute Proteins in Drosophila melanogaster. Mol. Cell. Biol..

[128] Kawamura Y., Saito K., Kin T., Ono Y., Asai K., Sunohara T., Okada T.N., Siomi M.C., Siomi H. (2008). Drosophila endogenous small RNAs bind to Argonaute 2 in somatic cells. Nature.

[129] Rozhkov N.V., Schostak N.C., Zelentsova E.S., Yushenova I.A., Zatsepina O.G., Evgen’ev M.B. (2013). Evolution and dynamics of small RNA response to a retroelement invasion in drosophila. Mol. Biol. Evol..

[130] Sarkies P., Selkirk M.E., Jones J.T., Blok V., Boothby T., Goldstein B., Hanelt B., Ardila-Garcia A., Fast N.M., Schiffer P.M. (2015). Ancient and novel small RNA pathways compensate for the loss of piRNAs in multiple independent nematode lineages. PLoS Biol..

[131] Desset S., Meignin C., Dastugue B., Vaury C. (2003). COM, a heterochromatic locus governing the control of independent endogenous retroviruses from Drosophila melanogaster. Genetics.

[132] Mohamed M., Dang N.T.M., Ogyama Y., Burlet N., Mugat B., Boulesteix M., Mérel V., Veber P., Salces-Ortiz J., Severac D. (2020). A Transposon Story: From TE Content to TE Dynamic Invasion of Drosophila Genomes Using the Single-Molecule Sequencing Technology from Oxford Nanopore. Cells.

[133] George P., Jensen S., Pogorelcnik R., Lee J., Xing Y., Brasset E., Vaury C., Sharakhov I.V. (2015). Increased production of piRNAs from euchromatic clusters and genes in Anopheles gambiae compared with Drosophila melanogaster. Epigenet. Chromatin.

[134] Wierzbicki F., Kofler R., Signor S. (2021). Evolutionary dynamics of piRNA clusters in Drosophila. Mol. Ecol..

